# A Proteomic Investigation of Hepatic Resistance to *Ascaris* in a Murine Model

**DOI:** 10.1371/journal.pntd.0004837

**Published:** 2016-08-04

**Authors:** Gwendoline Deslyper, Thomas J. Colgan, Andrew J. R. Cooper, Celia V. Holland, James C. Carolan

**Affiliations:** 1 Department of Biology, Maynooth University, Maynooth, County Kildare, Ireland; 2 School of Biological and Chemical Sciences, Queen Mary University of London, London, United Kingdom; 3 Department of Zoology, School of Natural Sciences, Trinity College Dublin, Dublin, Ireland; Queensland Institute of Medical Research, AUSTRALIA

## Abstract

The helminth *Ascaris* causes ascariasis in both humans and pigs. Humans, especially children, experience significant morbidity including respiratory complications, growth deficits and intestinal obstruction. Given that 800 million people worldwide are infected by *Ascaris*, this represents a significant global public health concern. The severity of the symptoms and associated morbidity are related to the parasite burden and not all hosts are infected equally. While the pathology of the disease has been extensively examined, our understanding of the molecular mechanisms underlying resistance and susceptibility to this nematode infection is poor. In order to investigate host differences associated with heavy and light parasite burden, an experimental murine model was developed utilising *Ascaris-*susceptible and -resistant mice strains, C57BL/6J and CBA/Ca, respectively, which experience differential burdens of migratory *Ascaris* larvae in the host lungs. Previous studies identified the liver as the site where this difference in susceptibility occurs. Using a label free quantitative proteomic approach, we analysed the hepatic proteomes of day four post infection C57BL/6J and CBA/Ca mice with and without *Ascaris* infection to identify proteins changes potentially linked to both resistance and susceptibility amongst the two strains, respectively. Over 3000 proteins were identified in total and clear intrinsic differences were elucidated between the two strains. These included a higher abundance of mitochondrial proteins, particularly those associated with the oxidative phosphorylation pathway and reactive oxygen species (ROS) production in the relatively resistant CBA/Ca mice. We hypothesise that the increased ROS levels associated with higher levels of mitochondrial activity results in a highly oxidative cellular environment that has a dramatic effect on the nematode’s ability to successfully sustain a parasitic association with its resistant host. Under infection, both strains had increased abundances in proteins associated with the oxidative phosphorylation pathway, as well as the tricarboxylic acid cycle, with respect to their controls, indicating a general stress response to *Ascaris* infection. Despite the early stage of infection, some immune-associated proteins were identified to be differentially abundant, providing a novel insight into the host response to *Ascaris*. In general, the susceptible C57BL/6J mice displayed higher abundances in immune-associated proteins, most likely signifying a more active nematode cohort with respect to their CBA/Ca counterparts. The complement component C8a and S100 proteins, S100a8 and S100a9, were highly differentially abundant in both infected strains, signifying a potential innate immune response and the importance of the complement pathway in defence against macroparasite infection. In addition, the signatures of an early adaptive immune response were observed through the presence of proteins, such as plastin-2 and dipeptidyl peptidase 1. A marked decrease in proteins associated with translation was also observed in both C57BL/6J and CBA/Ca mice under infection, indicative of either a general response to *Ascaris* or a modulatory effect by the nematode itself. Our research provides novel insights into the *in vivo* host-*Ascaris* relationship on the molecular level and provides new research perspectives in the development of *Ascaris* control and treatment strategies.

## Author Summary

*Ascaris* infection is a significant burden on the people who live in developing countries with infection being linked to poor hygiene and low socio-economic status. The parasite causes a range of symptoms, especially in children, which include both chronic morbidity, such as growth retardation, and acute outcomes, such as intestinal obstruction. Certain people tend to be more heavily infected than others, with those individuals experiencing worse morbidity. The understanding of the difference between susceptible and resistant people is an essential first step in the development of new therapies in order to eliminate this neglected parasitic disease. Using an established mouse model involving a susceptible and resistant strain, we aimed to gain insight into the host-*Ascaris* interaction at the hepatic interface and elucidate some of the molecular mechanisms potentially involved in resistance. A number of key intrinsic differences were determined between both strains including major differences in mitochondrial and ROS associated processes which may present the nematodes with differing oxidative conditions and explain the failure of the nematode to establish a successful parasitism in the resistant strain. In addition, we resolved signatures of the innate and early adaptive immune response and a major reduction in the proteins associated with translation in both strains under infection. Our findings need to be further explored, but could be the foundation for a better understanding of the mechanisms behind the differential parasite burden and in the future, potential new therapies for control.

## Introduction

Ascariasis is an important, widespread geohelminth disease of humans and pigs [1]. Over 800 million people are estimated to be infected with the causative agent, the human roundworm, *Ascaris lumbricoides* [2] and the equivalent in pigs, *Ascaris suum*, is equally ubiquitious [3]. The impact on children is particularly severe, with chronic morbidity such as growth retardation and diminished cognitive development being exhibited [4]. Transmission is linked to poor disposal of human waste leading to extensive contamination of the environment with long-lived, resistant eggs that embryonate under appropriate conditions of temperature and moisture [4].

The intensity of worm burden in the intestine of the host is a major determinant of the severity of disease [5]. Furthermore, the number of macroparasites a host carries is fundamental to our understanding of helminth parasite epidemiology [5]. Since the seminal work of Crofton [6], that characterized the frequency distribution of macroparasites as clumped or overdispersed, the ubiquity of what is now known as aggregation, has been demonstrated for a wide range of host-macroparasite systems [7]. Longitudinal field-based studies that measured the patterns of helminth re-infection in individual patients after the provision of anthelmintic treatment identified the phenomenon known as predisposition [8], whereby individuals demonstrated a degree of consistency in their patterns of re-infection and this was demonstrated from a range of endemic regions and geohelminth species [5]. Predisposition was also maintained over multiple rounds of chemotherapeutic treatment [9] and many years after a single round of treatment [10]. Aggregation and predisposition were also successfully modelled in outbred pigs infected with *A*. *suum* and were described as analogous to those of *A*. *lumbricoides* in humans [11].

The basis of this predisposition remains unknown [12] although it has been predicted that both exposure and host susceptibility are likely to influence the observed epidemiological patterns [5]. However, unravelling the relative contributions to aggregation and predisposition and hence susceptibility/resistance remains challenging for both ethical and logistical reasons [13]. As outlined by Keymer and Pagal [14], experimental manipulation utilising appropriate animal models is desirable, in tandem with human studies under field conditions, in order to study the multiple factors likely to be involved in predisposition.

*Ascaris* is a parasite that not only exists as an adult worm in the host intestine but also has a migratory pathway undertaken by its larvae, known as the hepato-trachaeal migration [15]. Symptoms occur during larval migration due to tissue damage [16] and the resultant pathology has been documented in the liver of both humans [17–19] and pigs [20–24]. Loeffler [25] described a transient or seasonal syndrome of pulmonary infiltrates, mild to marked respiratory symptoms and peripheral eosinophilia that he subsequently attributed to larval *Ascaris* in the lungs, termed Loeffler’s syndrome [26]. Pulmonary symptoms can be severe and life-threatening [27] and have also been documented in pigs [28].

Despite its global prevalence and sheer numbers of individuals it infects, ascariasis remains a classic neglected disease [1] and part of the explanation for this neglect, is because pigs as animal models are costly and laborious and we lack the versatility of inbred strains. The majority of model organisms that have been infected with *Ascaris* are so-called abnormal hosts, whereby the parasite does not complete its life-cycle, but manifests itself as the early migratory phase [29]. Furthermore, in the vast majority of such experimental systems, the basis of resistance or susceptibility to infection has not been clearly established [29]. In contrast, a convenient and repeatable mouse model for exploring the susceptibility to *Ascaris* during the early phase of infection has been developed [30–32].

C57BL/6J are uniquely susceptible to infection with the porcine ascarid, *A*. *suum*, and larval burdens recovered from the lungs of this strain, markedly exceed those recovered from similarly infected strains of which the most resistant is the CBA/Ca mouse [30]. These two strains therefore represent opposite response phenotypes [5], mimicking the extremes of predisposition detected in humans and pigs.

Subsequently, this model was utilised to assess the significance of inflammatory processes within the murine lung. Such responses mirrored larval intensity and it was concluded that the pulmonary inflammatory immune response was not prominently involved in primary protection of mice to *Ascaris* infection in the lungs [31]. The lack of support for a pulmonary mechanism led to the suggestion that a hepatic/post-hepatic factor, which varies between C57BL/6J and CBA/Ca mice, may play a critical role in the successful migration through host tissues [31]. Evidence in the form of a differential histopathological change in the liver between the two strains was observed, whereby the resistant strain, CBA/Ca demonstrated an earlier intense inflammatory response coupled with a more rapid tissue repair in the liver [32].

C57BL/6 and CBA/Ca mice have also been used as model organisms to study susceptibility and resistance to other helminth and protist parasites. For example, in the case of *Brugia malayi*, the parasites were cleared more rapidly from the blood stream in CBA/Ca mice compared to C57BL/6J mice [33]. Studies involving *Leishmania*, demonstrated that CBA mice were able to control infection with *Leishmania major*, but not an infection with *Leishmania amazonensis* [34]. C57BL/6 mice conversely were found to be resistant to *L*. *major* infection, but were, as was the case for the CBA mice, susceptible to *L*. *amazonensis* [35]. *Schistosoma mansoni* caused low grade pathology in C57BL/6 mice, but a more aggravated pathology in their CBA counterparts [36]. In the case of *Plasmodium berghei* ANKA, both CBA and C57BL/6 were susceptible to the development of cerebral malaria [37]. In short, it is clear that both strains respond differently when challenged by a diversity of parasitic infections. However, depending on the parasite species, the mouse strains will exhibit a difference in resistance and susceptibility.

The aims of the present study were to investigate the differences in the liver proteomes between the two mouse strains, one deemed to be susceptible, the other resistant, with both control and infected groups within each strain. To address these aims we employed high throughput quantitative mass spectrometry (MS) which is routinely used to identify and quantify thousands of proteins from highly complex samples. We specifically used label-free quantitative (LFQ) mass spectrometry [38] on total protein extracts from the left liver lobes of C57BL/6J and CBA/Ca mice with and without *Ascaris* infection sampled on day 4 post-infection (p.i.). The hepatic lobe and time-point choice were based on the study of Dold *et al*. [32], who demonstrated relatively high and equivalent larval numbers in C57BL/6J and CBA/Ca left lobes at day 4 p.i., a time point at which they also observed the onset of a differential inflammatory response to infection between the two mouse strains. LFQ-based proteomics is increasingly being used to investigate human pathogens and parasites including *Chlamydia trachomatis* [39], *Acinetobacter baumannii* [40] and *Plasmodium vivax*- infected and uninfected erythrocytes [41] and is providing unprecedented insight into both specific and broad level interactions between the infectious agent and its host.

## Materials and Methods

### Ethics statement

The samples obtained for this study were part of a project authorised by the Health Products Regulatory Authority (HPRA), the competent authority that regulates scientific animal research in Ireland in accordance with Directive 2010/63/EU and its Irish transposition, SI No 543 of 2012 (Project Authorisation ID AE19136/P008 ID; Case Reference 7015826). In addition, this project was ethically approved by the TCD Animal Research Ethics Committee (AREC) prior to HPRA submission and approval.

### Sample preparation

Both C57BL/6J and CBA/Ca mice (Harlan laboratories) were infected with 1000 embryonated ova of *A*. *suum* and euthanized using cervical dislocation on day four post infection (p.i.). At this time point, five animals were euthanised from all four groups: C57BL/6J infected, C57BL/6J control, CBA/Ca infected and CBA/Ca control [42]. The livers were extracted and each lobe was snap frozen separately using liquid nitrogen and stored at -80°C until required.

To confirm a differential larval burdens in susceptible and resistant strains, post-mortems of 5 infected mice from each strain were conducted on day 7 post-infection. Living *Ascaris* larvae were recovered from the lungs of each mouse by means of the modified Baermann technique [30]. A pellet of the isolated viable larvae was suspended in a 5 ml solution of 0.9% saline and 6% formalin. Prior to larval counts the 5 ml solutions were agitated to ensure a homogeneous distribution of larvae within the sample. Larval counts were recorded from lung samples by means of pipetting 2 mls of solution into the chamber of a nematode counting slide (Chalex corporation)[32]. The number of larvae in the gridded area, which represented 1 ml, was counted under X40 magnification. The number of larvae in a 1 ml solution was multiplied by the total volume in order to estimate the number of larvae in each lung sample.

The left lobes of day four p.i. were homogenized in 6M urea, 2M thiourea, supplemented with a protease inhibitor cocktail (Roche, Complete Mini). Samples were centrifuged for 5 min at 11,200 × g to pellet any cellular debris. The supernatant was then removed and quantified using the Qubit^™^ protein quantification system (Invitrogen), following the manufacturer’s instructions. Three independent biological replicates for each group were analysed in this study. 75 μg of each sample was precipitated using the 2D Clean-Up Kit (GE HealthCare), following the manufacturer’s instructions. The resulting protein pellet was resuspended in 6M urea, 2M thiourea, 0.1 M Tris-HCl, pH 8.0. 50mM ammonium bicarbonate was added to each sample and proteins were reduced with 0.5M dithiothreitol (DTT) at 56°C for 20 min and alkylated with 0.55M iodoacetamide (IAA) at room temperature for 15 min, in the dark. 1 μl of a 1% w/v solution of Protease Max Surfactant Trypsin Enhancer (Promega) and 1 μg of Sequence Grade Trypsin (Promega) was added to give a protein:trypsin ratio of 75:1. The protein/trypsin mixture was incubated at 37°C for 18 hours. Digestion was terminated by adding 1 μl of 100% trifluoroacetic acid (Sigma Aldrich) and incubation at room temperature for 5 min. Samples were centrifuged for 10 min at 13,000 × g and a volume equivalent to 40 μg of pre-digested protein was removed and purified for mass spectrometry using C18 Spin Columns (Pierce), following the manufacturer’s instructions. The eluted peptides were dried using a SpeedyVac concentrator (Thermo Scientific Savant DNA120) and resuspended in 2% v/v acetonitrile and 0.05% v/v trifluoroacetic acid (TFA). Samples were sonicated for 5 min to aid peptide resuspension followed by centrifugation for 5 min at 16,000 × g. The supernatant was removed and used for mass spectrometry.

### Mass spectrometry

1 μg of each digested sample was loaded onto a QExactive (ThermoFisher Scientific) high-resolution accurate mass spectrometer connected to a Dionex Ultimate 3000 (RSLCnano) chromatography system. The peptides were separated by a 2% to 40% gradient of acetonitrile on a Biobasic C18 Picofrit column (100mm length, 75mm ID), using a 120 minute reverse-phase gradient at a flow rate of 250nL min^-1^. All data were acquired with the mass spectrometer operating in automatic data dependent switching mode. A full MS scan at 140,000 resolution and a range of 300–1700 m/z was followed by an MS/MS scan, resolution 17,500 and a range of 200–2000 m/z, selecting the 15 most intense ions prior to MS/MS.

Protein identification and LFQ normalisation of MS/MS data was performed using MaxQuant v1.5.0.8 (http://www.maxquant.org) following the general procedures and settings outlined in [43]. The Andromeda search algorithm [44] incorporated in the MaxQuant software was used to correlate MS/MS data against the SWISS-PROT database for *Mus musculus* [45](16,773 entries, downloaded May 2015) and a contaminant sequence set provided by MaxQuant. The following search parameters were used: first search peptide tolerance of 20 ppm, second search peptide tolerance 4.5 ppm with cysteine carbamidomethylation as a fixed modification and N-acetylation of protein and oxidation of methionine as variable modifications and a maximum of two missed cleavage sites allowed. False Discovery Rates (FDR) were set to 1% for both peptides and proteins and the FDR was estimated following searches against a target-decoy database. LFQ intensities were calculated using the MaxLFQ algorithm [46] from razor and unique peptides with a minimum ratio count of two peptides across samples. Peptides with minimum length of seven amino acids were considered for identification and proteins were only considered identified when more than one unique peptide for each protein was observed.

### Data analysis

Perseus v.1.5.0.8 (www.maxquant.org/) was used for data analysis, processing and visualisation. Normalised LFQ intensity values were used as the quantitative measurement of protein abundance for subsequent analysis. The data matrix was first filtered for the removal of contaminants and peptides identified by site. LFQ intensity values were log_2_ transformed [47] and each sample was assigned to its corresponding group (C57BL/6J control and infected; CBA/Ca control and infected). Proteins not found in two out of three replicates in at least one group were omitted from the analysis. A data-imputation step was conducted to replace missing values with values that simulate signals of low abundant proteins [48] chosen randomly from a distribution specified by a downshift of 2.6 times the mean standard deviation (SD) of all measured values and a width of 0.37 times this SD.

Two sample t-tests were performed for all relevant comparisons using a cut-off of p<0.05 on the post imputated dataset to identify statistically significant differentially abundant (SSDA) proteins. Volcano plots were generated in Perseus by plotting negative log p-values on the y-axis and log_2_ fold-change values on the x-axis for each pair-wise comparison to visualise changes in protein expression. The ‘categories’ function in Perseus was utilized to highlight and visualise the distribution of various pathways and processes on selected volcano plots. Normalised intensity values were used for a principal component analysis (PCA). Exclusively expressed proteins (those that were uniquely expressed or completely absent in one group) were identified from the pre-imputation dataset and included in subsequent analyses. Hierarchical clustering was performed on Z-score normalised intensity values for all differentially abundant proteins by clustering both samples and proteins using Euclidean distance and complete linkage.

Gene ontology (GO) mapping was also performed in Perseus using the UniProt gene ID for all identified proteins to query the Perseus annotation file (downloaded January 2015) and extract terms for biological process, molecular function, Kyoto Encyclopaedia of Genes and Genomes (KEGG) name, KEGG pathway, protein family (pfam) and InterPro. GO and KEGG term enrichment analysis was performed on the major protein clusters identified by hierarchical clustering using a Fisher’s exact test (a Benjamini-Hochberg corrected FDR of2%) for enrichment in Uniprot Keywords, gene ontology biological process (GOBP), gene ontology cellular component (GOCC) and KEGG (FDR <2%). The Search Tool for the Retrieval of INteracting Genes/Proteins (STRING) [49] v10 (http://string-db.org/) was used to map known and predicted protein:protein interactions. UniProt gene lists (extracted from Perseus) were inputted and analysed in STRING using the medium to high confidence (0.5–0.7) setting to produce interactive protein networks for each group in all comparisons. GO term enrichment analyses for biological process, molecular function and cellular compartment were then conducted to identify potential pathways and processes that warranted further analysis. Such pathways were examined using the KEGG pathway analysis (http://www.kegg.jp/kegg/tool/map_pathway2.html) [50, 51], using the ‘KEGG Mapper—Search&Color Pathway’ tool. The equivalent KEGG identifiers were obtained using the UniProt ‘Retrieve/ID mapping’ function (http://www.uniprot.org/uploadlists/) with the organism set to *M*. *musculus* (mmu). Retrieved KEGG IDs were used to identify the most represented pathways. The MS proteomics data and MaxQuant search output files have been deposited to the ProteomeXchange Consortium [52] via the PRIDE partner repository with the dataset identifier PXD003555.

## Results

The mean larval burden in the lungs of the C57BL/6J mice (n = 5) was 188 ±SD 78.7 and the mean larval burden for CBA/Ca mice (n = 5) was 12 ±SD 8.5 confirming the resistant and susceptible phenotype in the mice used in our experiment.

**Fig 1:**
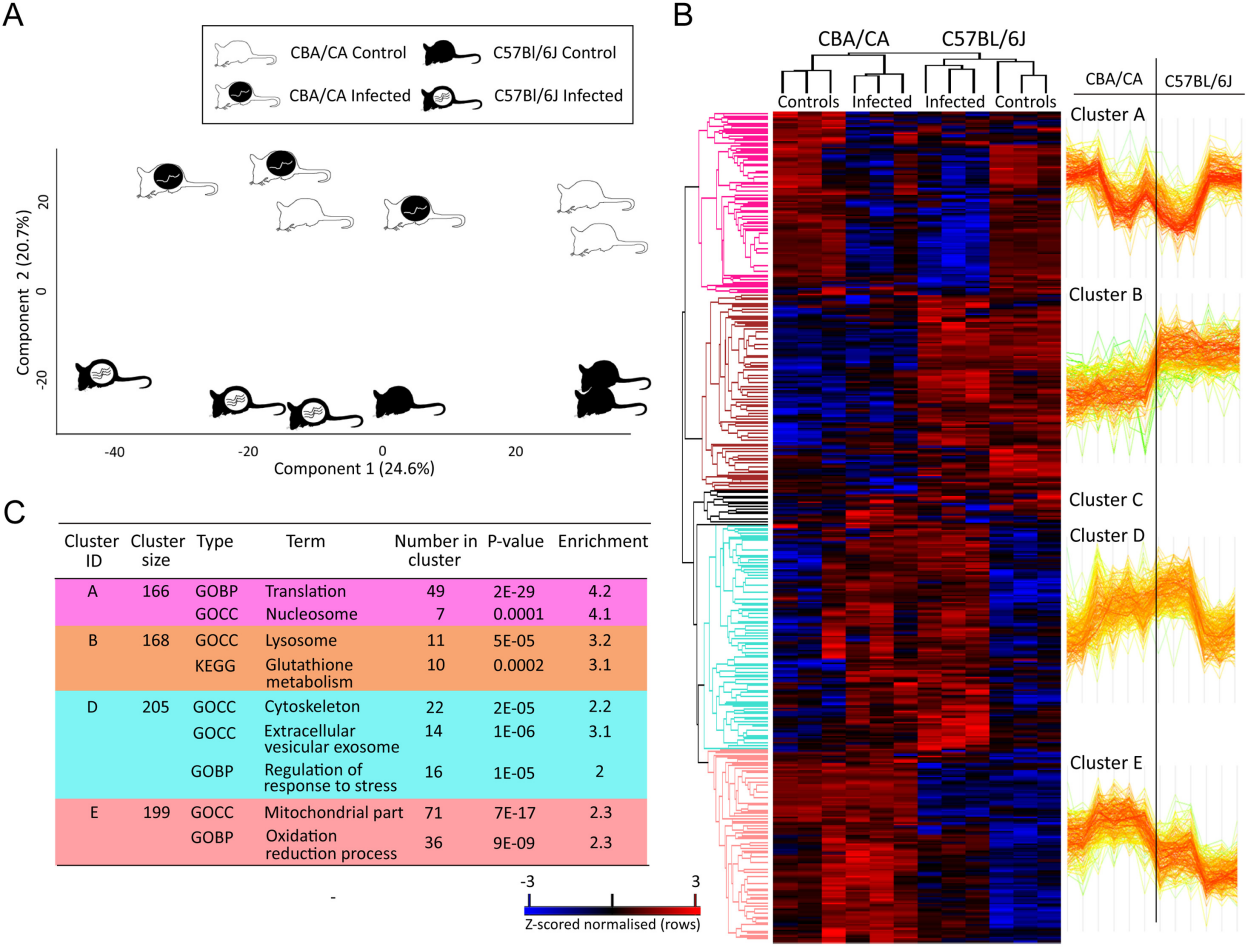
Label free quantitative proteomic analysis of liver left lobes of C57BL/6J and CBA/Ca mice with and without *Ascaris* infection. (A) Principal component analysis (PCA) of the left lobe liver proteomes of C57BL/6J and CBA/Ca mice with and *without* Ascaris infection. A clear distinction can be observed between the strains and the distinction between C57BL/6J infected and controls is greater than that for CBA/Ca infected and controls. (B) Two-way unsupervised hierarchical clustering of the median protein expression values of all statistically significant differentially abundant proteins. Hierarchical clustering (columns) resolved four distinct clusters comprising the three replicates from their original sample groups and five protein cluster (rows) based on expression profile similarities. (C) The table shows a selection of significantly enriched GOBP and GOCC terms (Fisher’s exact test, FDR < 2%) in each of the identified protein clusters.

LFQ MS was performed on three replicates originating from the liver left lobes of C57BL/6J and CBA/Ca mice with and without *Ascaris* infection. 3,145 proteins were identified initially of which 2,307 remained after filtering and processing (S1 Dataset). Proteins, exclusive to or absent from an individual sample were included in subsequent analyses as proteins of significant interest (S1 Table). A principal component analysis (PCA) performed on all filtered proteins (Fig 1A) distinguished the C57BL/6J and CBA/Ca samples indicating a clear intrinsic difference between the two strains. Although the C57BL/6J infected and controls are well resolved there is some overlap within CBA/Ca infected and control groups, indicating that the response to *Ascaris* in C57BL/6J is perhaps more pronounced.

Hierarchical clustering of z-score normalised intensity values for all significantly differentially abundant proteins (n = 194) resolved the three replicates of each sample group (Fig 1B). In addition, one minor and four major protein clusters were identified: the latter comprising C57BL/6J and CBA/Ca abundant proteins (Cluster A), C57BL/6J control and infected proteins (Cluster B); predominantly C57BL/6J and CBA/Ca infected abundant proteins (Cluster D) and CBA/Ca control and infected abundant proteins (Cluster E). GO and KEGG term enrichment analysis identified key biological processes enriched within each cluster (S2 Table; summarised in Fig 1C) and included translation and nucleosome (Cluster A); lysosome and glutathione metabolism (Cluster B); cytoskeleton, regulation of response to stress and extracellular vesicular exosome (Cluster D) and mitochondrial part and oxidation-reduction process (Cluster E).

### Differential abundance and interaction network analysis

Performing two sample t-tests resulted in the identification of SSDA proteins both between and within strains (S2 Dataset). These differentially abundant proteins, together with proteins uniquely expressed in each group, were analysed using the interaction network analysis software, STRING, to identify biological pathways and processes over-represented in a particular group. Biological processes and pathways identified by STRING were investigated further for their representation within the differentially abundant protein dataset and displayed on volcano plots using the ‘categories’ function in Perseus to highlight proteins involved in selected biological processes.

### C57BL/6J infected compared to C57BL/6J control

**Fig 2:**
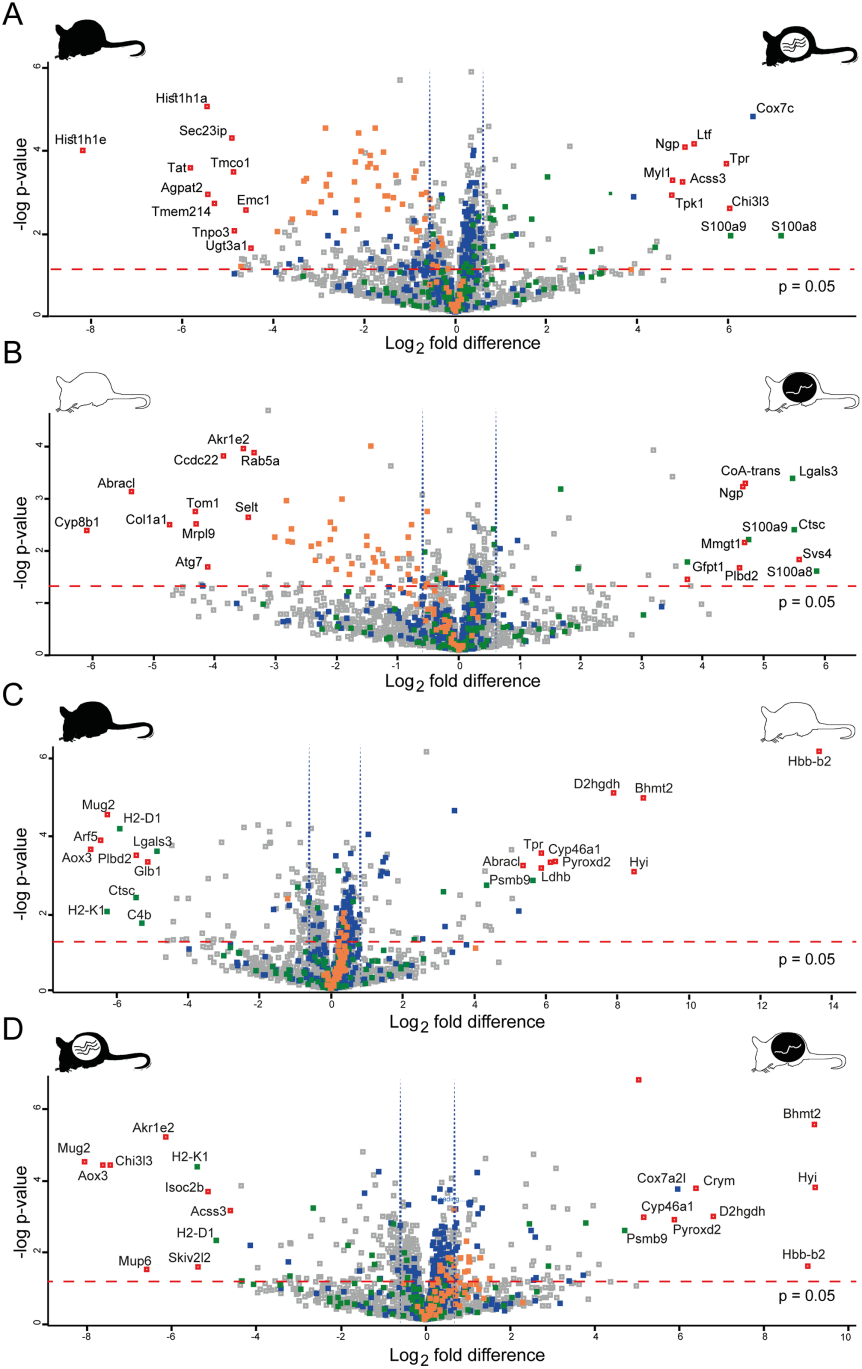
Volcano plots of all identified proteins based on relative abundance differences between C57BL/6J and CBA/Ca mice with and without *Ascaris*. Volcano plots showing the distribution of quantified proteins according to p value (−log_10_ p-value) and fold change (log_2_ mean LFQ intensity difference). Proteins above the line are considered statistically significant (p-value <0.05) and those to the right and left of the vertical lines indicate relative fold changes ≥ 1.5. The top 20 differentially abundant proteins are annotated and all proteins associated with the mitochondria (blue), immune system (green) and translation (orange) are highlighted for **(A)** C57BL/6J infected and control, **(B)** CBA/Ca infected and control mice, **(C)** CBA/Ca control and C57BL/6J control and **(D)** CBA/Ca infected and C57BL/6J infected mice.

**Fig 3:**
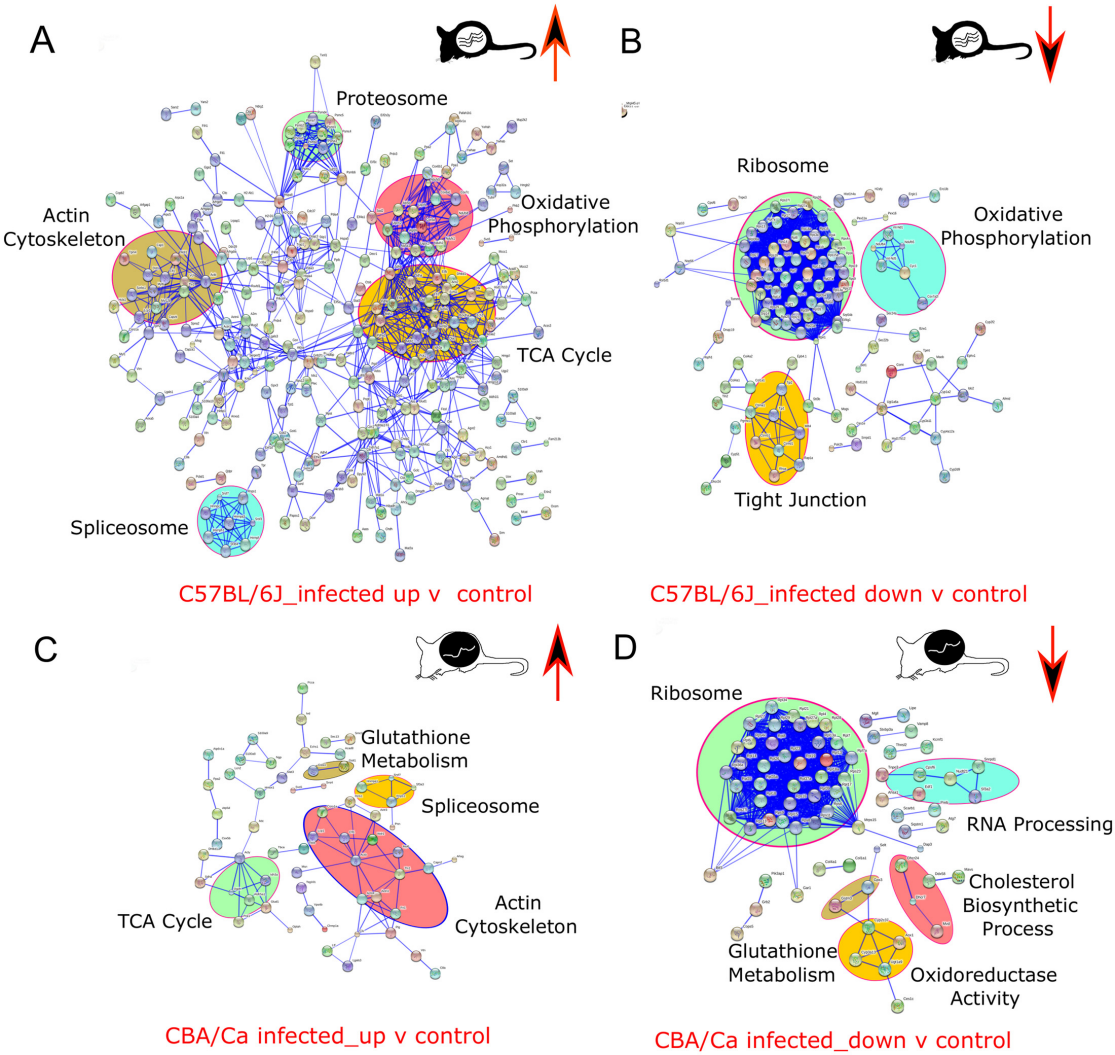
Interaction network analysis of up regulated proteins in C57BL/6J and CBA/Ca mice with and without *Ascaris* infection. Protein interaction information was obtained from the STRING database using gene lists extracted for statistically significant differentially abundant (SSDA) proteins from pair wise t-tests (p< 0.05). Each node represents a protein and each connecting line represents an interaction, the extent of evidence for which is represented by the width of the line. Statistically enriched KEGG and biological process Gene Ontology (GO) descriptors were examined to identify clusters of proteins enriched within SSDA protein of increased abundance for **(A)** C57BL/6J infected in comparison to C57BL/6J controls, **(B)** C57BL/6J controls in comparison to C57BL/6J infected, **(C)** CBA/Ca infected in comparison to CBA/Ca controls and **(D)** CBA/Ca controls in comparison to CBA/Ca infected mice.

Two sample t-tests (p<0.05) on the post-imputation dataset identified 479 SSDA proteins between C57BL/6J infected and control with the log_2_ fold change ranging from -8.2 to 7.2 (Table 1). The 20 most differentially abundant proteins for both groups are displayed in Fig 2A. The most abundant proteins in C57BL/6J controls were histone H1.4, tyrosine aminotransferase, and histone H1.1. In comparison, for C57BL/6J infected samples, the most abundant proteins consisted of two S100 proteins (protein S100-A8 and protein S100-A9) and cytochrome c oxidase subunit 7C protein. STRING analysis identified enrichment of terms associated with the proteasome, the mitochondrion, RNA splicing and actin cytoskeleton in C57BL/6J infected individuals (Fig 3A) whereas enriched terms were identified for translation in the C57BL/6J controls (Fig 3B).

**Table 1:** Numbers of statistically significant differentially abundant (SSDA) proteins (t-tests; p<0.05) identified among CBA/Ca and C57BL/6J infected and uninfected samples. The number of proteins before and after imputation, the range of Log_2_ fold differences and the number of proteins associated with well represented KEGG or gene ontology processed or pathways are given. Numbers in parenthesis represent the total number of proteins identified within each process. OXPHOS: oxidative phosphorylation, FC: fold change.

			Protein Processes				
T-test Comparison	Number of proteins*	Range of FC Difference	Mitochondria (324)	OXPHOS (80)	Immune system (106)	Ribosomes (73)	Iron ion binding (71)
C57BL/6J infected: C57BL/6J control	456/479	-8.2/7.2	81	22	20	48	21
CBA/Ca infected: CBA/Ca control	169/193	-6.1/5.9	17	5	14	35	5
CBA/Ca control: C57BL/6J control	320/354	-6.7/13.6	72	25	14	9	11
CBA/Ca infected: C57BL/6J infected	374/410	-8.1/9.2	78	30	15	16	14

* pre-imputation/post imputation

### CBA/Ca infected compared to CBA/Ca control

Two sample t-tests on the post-imputation dataset identified 193 SSDA proteins (log_2_ fold change range: -6.1 to 5.9; Table 1) with the 20 most differentially abundant proteins displayed in Fig 2B. The most abundant proteins in CBA/Ca infected mice were: protein S100-A8, seminal vesicle secretory protein 4, and dipeptidyl peptidase 1. For CBA/Ca control mice the most SSDA proteins included: 7-alpha-hydroxycholest-4-en-3-one 12-alpha-hydroxylase, costars family protein ABRACL, and collagen alpha-1(I) chain. STRING analysis revealed that CBA/Ca infected mice had an overrepresentation of spliceosome and actin cytoskeleton proteins in comparison to control samples (Fig 3C). As observed in the C57BL/6J mice, terms associated with translational proteins were enriched in addition to RNA processing and glutathione metabolism in CBA/Ca control mice with respect to their infected counterparts (Fig 3D).

### CBA/Ca control compared to C57BL/6J control

Two sample t-tests (p<0.05) on the post-imputation dataset identified 354 SSDA proteins respectively and the Log_2_ fold changes ranged from -6.7 to 13.6 (Table 1). The top 20 most SSDA proteins are shown in Fig 2C. The most abundantly expressed proteins observed in CBA/Ca control samples are haemoglobin subunit beta-2, S-methylmethionine, and putative hydroxypyruvate isomerase. The log_2_ fold change for haemoglobin subunit beta-2 was the highest observed in the dataset, being 13.6. For C57BL/6J controls, the most abundantly expressed proteins are aldehyde oxidase 3, ADP-ribosylation factor 5, and H-2 class I histocompatibility antigen K-B alpha chain. Two clusters were observed in the CBA/Ca control samples representing ribosomal and oxidative phosphorylation biological processes (S1A Fig). However STRING analysis failed to identify any well populated clusters in C57BL/6J compared to CBA/Ca control (S1B Fig).

### CBA/Ca infected compared to C57BL/6J infected

Two sample t-tests (p<0.05) on the post-imputation dataset identified 410 SSDA proteins, respectively and the log_2_ fold changes ranged from -8.1 to 9.2 (Table 1). The 20 most SSDA proteins are displayed in Fig 2D. The most abundant proteins in CBA/Ca infected compared to C57BL/6J were found to be: putative hydroxypyruvate isomerase, S-methylmethionine, haemoglobin subunit beta-2. The most abundant proteins present in C57BL/6J infected compared to their CBA/Ca counter parts were: murinoglobulin-2, aldehyde oxidase 3, and chitinase-3-like protein 3. STRING analysis resolved clusters with enriched terms for oxidative phosphorylation and translation in CBA/Ca infected mice (S1C Fig). Terms associated with the glutathione metabolism are enriched in C57BL/6J infected mice with respect to CBA/Ca infected mice (S1D Fig).

### Enriched processes and pathways

Proteins involved in the processes and pathways of interest (identified in Perseus and STRING) were displayed on volcano plots arising from the 2-way t-tests to give an indication of expression profile for all significant and non-significant representatives. The five processes that were consistently overrepresented and/or differentially abundant and selected for further analysis were mitochondria, electron transfer chain, immune system, ribosome and iron ion binding (Table 1). KEGG analysis was also used to provide a protein centric view for selected pathways (S2–S4 Figs).

### Mitochondria and associated processes

Mitochondrial associated proteins were consistently differentially abundant among all samples. Of the 2,307 proteins identified and reported here, 324 were associated with the mitochondrion. A higher abundance of mitochondrial proteins was generally observed in CBA/Ca mice both with and without infection (Table 1; Fig 1C) in comparison to C57BL/6J mice, although an increase in mitochondrial protein abundances was observed for both strains under infection (Fig 2). GO analysis in Perseus resolved 80 oxidative phosphorylation (OXPHOS) terms, including electron transfer chain (ETC) complex I to V proteins, the most differentially abundant of which were cytochrome c oxidases (cox7c and cox7a2). CBA/Ca control and infected mice had a higher abundance of proteins involved in the OXPHOS pathway, when compared to C57BL/6J (S2A Fig). Although there was a significant upregulation of OXPHOS proteins in C57BL/6J infected mice when compared to their controls, the extent of increase was less than that for CBA/Ca. KEGG analysis also indicated that there was a generally lower abundance of ETC proteins in C57BL/6J infected mice, compared to their CBA/Ca counterparts. However Complex I proteins, were less abundant in C57BL/6J infected mice, when compared to their controls (S2A Fig).

Proteins of the tricarboxylic acid (TCA) cycle were intrinsically more abundant in CBA/Ca control mice, when compared to their C57BL/6J counterparts. This difference between the two strains was observed also under infection. When infected, both mouse strains had increased abundance of these proteins. However, C57BL/6J infected mice had higher abundances for the entire TCA cycle (S3 Fig).

### Translation

A clear change in the abundance of proteins involved in the translational process during infection was observed. Ribosomal proteins were more abundant in both CBA/Ca controls and infected with respect to their C57BL/6J counterparts (S1A and S1C Fig). Under infection however, both CBA/Ca and C57BL/6J mice had significantly lower abundances for many ribosomal proteins (Fig 2). KEGG analysis further confirmed this finding demonstrating that CBA/Ca had more intrinsically abundant ribosomal proteins than C57BL/6J, a difference that became more pronounced under infection (S2B Fig).

### Immune system and response to stress

GO and KEGG term enrichment analysis indicated that there were very few immune associated pathways respondent to infection. Of these, 20 immune associated proteins were differentially abundant (relative fold difference > 1.5) between infected and control samples (Table 2). Eight proteins: s100-A8; s100-A9; dipeptidyl peptidase 1; coronin-1A; galectin; vitronectin; moesin and plasminogen were more abundantly expressed in both C57BL/6J and CBA/Ca infected mice with respect to their uninfected counterparts indicating a potential conservation of response to *Ascaris* in both mice strains. The majority of SSDA immune proteins were observed in C57BL/6J *Ascaris*-infected mice (Table 2). A number of immune proteins demonstrated higher intrinsic abundances in C57BL/6J compared to CBA/Ca (in both infected and uninfected comparisons) and included dipeptidyl peptidase 1, complement C4-B, galectin-3 and the MHC class I molecules H2-D1 and H2-K1 (Fig 2C and 2D).

**Table 2:** Statistically significant differentially abundant (SSDA) proteins associated with immune processes. Log_2_ fold differences from two pairwise t-tests (p < 0.05) are given for (i) CBA: CBA/Ca infected and CBA/CaC57 control and (ii) C57: C57BL/6J infected and C57BL/6J control. The number of immune associated proteins and relative fold change differences are for the majority greater in C57BL/6J than CBA/Ca *Ascaris*-infected mice. Immune functions were retrieved by searching the UniProt ID on the UniProt Knowledgebase (http://www.uniprot.org/).

Gene names	Protein names	Uniprot ID	T-test Comparison	Log2 fold Difference	Immune Function
S100a8	Protein S100-A8	P27005	C57; CBA	7.2; 5.9	Innate immune response; Leukocyte migration in inflammatory response; Neutrophil chemotaxis; Positive regulation of inflammatory response; Autophagy; Positive regulation of apoptotic signalling
S100a9	Protein S100-A9	P31725	C57; CBA	6.1; 4.7	Actin cytoskeleton reorganisation; Leukocyte chemotaxis; Positive regulation of inflammatory response; Positive regulation of blood coagulation; Autophagy
Ctsc	Dipeptidyl peptidase 1	P97821	CBA; C57	5.5; 1.3	T cell mediated cytotoxicity; Positive regulation of apoptotic signalling pathway
Lgals3	Galectin-3	P16110	CBA; C57	5.5; 1.7	IgE binding; Eosinophil/macrophage/monocyte/neutrophil Chemotaxis; Innate immune response
Anxa1	Annexin A1	P10107	C57	4.4	Granulocyte/monocyte chemotaxis; Phagocytosis; Negative regulation of T-helper 2 cell differentiation; Neutrophil homeostasis; Positive regulation of T-helper 1 cell differentiation; Positive regulation of interleukin-1 and 2 production; Positive regulation of neutrophil apoptotic process; Hepatocyte differentiation
Coro1a	Coronin-1A	O89053	CBA; C57	3.7; 3.0	Leukocyte chemotaxis; Natural killer cell degranulation Phagocytosis; Cellular response to interleukin-4; Positive regulation of T cell activation and proliferation
Lcn2	Neutrophil gelatinase-associated lipocalin	P11672	C57	3.4	Cellular response to hydrogen peroxide; Cellular response to interleukin-1; Innate immune response
Vtn	Vitronectin	P29788	C57 CBA	2.0 1.7	Immune response; Positive regulation of smooth muscle cell migration; Positive regulation of receptor-mediated
Ilf3	Interleukin enhancer-binding factor 3	Q9Z1X4	CBA	2.0	Defense response to virus
Fth1	Ferritin heavy chain	P09528	C57	1.1	Immune response; Negative regulation of cell proliferation; Negative regulation of necrotic cell death
Ahsg	Alpha-2-HS-glycoprotein	P29699	C57	0.9	Acute-phase response; Positive regulation of phagocytosis; Regulation of inflammatory response
Irak4	Interleukin-1 receptor-associated kinase 4	Q8R4K2	C57	0.9	Neutrophil mediated immunity; JNK cascade; Positive regulation of I-kappaB kinase/NF-kappaB signalling
Lgals1	Galectin-1	P16045	C57	0.8	Positive regulation of I-kappaB kinase/NF-kappaB signalling; T cell costimulation
Msn	Moesin	P26041	C57; CBA	0.8; 0.6	Leukocyte cell-cell adhesion; Leukocyte migration
Anxa2	Annexin A2	P07356	C57	0.8	Angiogenesis; Fibrinolysis; Positive regulation of fibroblast proliferation
Plg	Plasminogen	P20918	C57 CBA	0.8 0.6	Mononuclear cell migration; Negative regulation of angiogenesis; Tissue regeneration
Pafah1b1	Platelet-activating factor acetylhydrolase IB subunit alpha	P63005	C57	0.7	Actin cytoskeleton organization; Negative regulation of JNK cascade; Positive regulation of cytokine-mediated signaling; Regulation of microtubule motor activity
Serpinf2	Alpha-2-antiplasmin	Q61247	C57	0.6	Acute-phase response; Positive regulation of stress fibre assembly; Regulation of blood vessel size by renin-angiotensin; Negative regulation of plasminogen activation
Hpx	Hemopexin	Q91X72	C57	0.6	Heme metabolic process; Positive regulation of immunoglobulin production; Positive regulation of interferon-gamma-mediated signalling; Positive regulation of response to interferon
C8a	Complement component C8 alpha chain	Q8K182	C57; CBA	0.6; 0.6	Complement activation; Cytolysis

16 proteins associated with the biological process term “response to stress” were enriched in the shared “response to infection” cluster (Cluster D on Fig 1B). Many of these proteins are also associated with the cytoskeleton and cytoskeletal organisation, terms that were also shown to be significantly enriched within this “infected” cluster (S2 Table; Fig 1C).

### Role of glutathione metabolism in resistance

Glutathione is known to reduce the reactive oxygen species (ROS), H_2_O_2_ to H_2_O. Because of its ROS reducing capacities, it was investigated in this study. KEGG analysis identified an increase in glutathione metabolic proteins of C57BL/6J control samples, compared to their CBA/Ca counterparts (S4 Fig). This inherent difference was also observed when comparing both strains under infection. Additionally, there was an increase in abundance of glutathione metabolism proteins in C57BL/6J mice when infected, compared to their controls. This increase was less pronounced in CBA/Ca mice.

## Discussion

With 800 million people worldwide infected with *Ascaris* [2], improving our understanding of the underlying biological pathways involved with susceptibility to infection is required. Currently, ascariasis control consists primarily of the provision of anthelmintic drugs, such as albendazole or mebendazole [53], however, reinfection can occur rapidly after treatment [54]. Furthermore, the presence of adult worms in the intestine contributes to significant chronic morbidity and, in some cases, more acute complications [4]. Therefore, investigation of the role of the liver—a key organ in *Ascaris* larval migration, and likely attrition and consequent establishment of adult worms in the intestine could prove fruitful [29]. The liver has previously been identified as the organ of interest to investigate susceptibility and resistance to *Ascaris* infection [31, 32]. To understand the difference in susceptibility and resistance to heavy infection, we have performed a proteomic analysis of the liver of two different mouse strains, C57BL/6J and CBA/Ca, models for susceptibility (heavy larval burden) and resistance (light larval burden), respectively. The difference in susceptibility was confirmed by assessing larval burdens in the lungs C57BL/6J and CBA/Ca mice on day 7 p.i. Previous studies have demonstrated a distinct, repeatable difference in *A*. *suum* larval burden in the lungs of susceptible C57BL/6J and resistant CBA/Ca inbred mice on day 7 post-infection [30, 32, 55]. Although the numbers observed were very similar to those described in these studies, a clearer divergence in larval numbers was observed between both strains in the present experiment.

Using quantitative mass spectrometry, several pathways and individual proteins, which may contribute to these observed differences in the burden of infection, were identified. A clear difference in the proteomes of both strains was observed (Fig 1A) and intrinsic differences identified between the inbred strains, unrelated to *Ascaris* infection, which may underpin susceptibility and resistance, and are also significant for other branches of biomedical research using these particular mice strains.

### Mitochondrial differences potentially determine resistance or susceptibility

Parasite infection can place additional stresses on the physiological processes of the host. One of the major findings of this study was the higher abundance of proteins involved in mitochondrial processes in the CBA/Ca mouse strain, both with and without infection. These processes include OXPHOS and the TCA cycle, which were significantly enriched in the CBA/Ca proteome in comparison to their C57BL/6J counterparts. These intrinsic differences may represent a significant difference between both strains in terms of the susceptibility to *Ascaris* infection. The mitochondria are the main source of ROS, which are produced as by-products in the OXPHOS pathway as part of cellular respiration [56, 57]. ROS have a vast range of biological functions and play a role in defence against parasites and pathogens, apoptosis, cell survival, cell growth, proliferation, differentiation and many other signalling pathways [57, 58]. Higher ROS production in CBA/Ca mice may generate a prohibitive oxidative environment for *Ascaris* within the murine liver and explain the decrease in observable migration to the lungs by *Ascaris* from the CBA/Ca strain [32]. Our results support previous findings that CBA/Ca mice have higher superoxide production and have a higher tolerance to ROS than their C57BL/6 counterparts [59, 60]. Staecker *et al*. [60] found that the mouse cochlea of the C57BL/6J strain showed an early-onset hearing loss pathology compared to CBA/Ca mice, potentially linked to increased levels of free radicals present in the cochlea. This increase was attributed to the *adult hearing loss* (*Ahl*) gene, first identified by Johnson *et al*. [61], which is thought to cause a decrease in protective antioxidant enzymes in C57BL/6J mice. Taken together, these studies suggest that CBA/Ca mice not only produce higher endogenous levels of ROS and protective antioxidants but can tolerate ROS levels that could potentially affect an invading parasite.

Under infection, both strains exhibited higher abundances of OXPHOS and TCA cycle proteins indicating a general stress response to *Ascaris* infection. Although the increase in abundance was generally more pronounced in C57BL/6J mice, when compared to their corresponding controls, infected CBA/Ca mice had higher overall abundances representing the higher endogenous levels of mitochondrial proteins in CBA/Ca control mice. Interestingly, C57BL/6J infected mice had lower abundances of complex I proteins specifically in comparison to their controls. Complex I is an important site of superoxide production (together with complex III) [62] and has been linked with a number of diseases spanning early childhood (Leigh disease) [63] to adulthood (Parkinson’s and Alzheimer’s disease) [62]. Additionally, quinone-binding inhibitors, which inhibit complex I, were shown to increase ROS production [62]. TCA cycle proteins showed a similar pattern of abundance changes amongst samples, with higher intrinsic abundances observed in CBA/Ca mice and increased abundances observed under infection. However, C57BL/6J infected mice displayed higher abundances of TCA-associated proteins under infection than CBA/Ca infected mice, compared to their controls. The TCA cycle takes place in the mitochondria [64] and results in the production of the reduced co-enzyme nicotinamide adenine dinucleotide hydride (NADH), which contributes electrons to the OXPHOS pathway. Thus, it seems that in response to infection both mice strains increase OXPHOS activity.

The differences in mitochondrial protein abundance and presumed ROS levels between the susceptible and resistant strains can be explained not only by intrinsic basal differences but also the extent of parasitic interaction within each strain. Based on the significant reduction in nematode numbers that reach the lungs in CBA/Ca mice [31] one can assume that the CBA/Ca-*Ascaris* interaction represents a far less compatible host-parasite interaction in comparison to the C57BL/6J-*Ascaris* interaction. Nematodes in C57BL/6J livers seem capable of successful establishment within their hosts and in doing so, modulate host defence pathways and responses to allow for completion of the parasite life-cycle. *Ascaris* secrete a plethora of proteins in their excretory/secretory fluid with potential immunomodulatory and antioxidant defence functions [65–67]. The first complete predicted secretome was generated during the *Ascaris suum* genome project identifying 775 predicted secretory proteins with a rich number of peptidases for the migration through host tissues, as well as products potentially involved in the evasion or modulation of host defences [68]. In addition, proteomic analyses of the excretory/secretory products of larval and adult *Ascaris* have characterised heat shock proteins, ABA-1, proteases, serpins, chitinases and a suite of individual proteins that have been implicated in immune evasion and modulation and have been suggested to play a role in parasite survival [69]. It is not unreasonable to postulate that *Ascaris* also secrete proteins that reduce host mitochondrial processes and ROS production specifically. Modulation of host ROS production has been widely reported for a number of taxonomically diverse parasites, including the liver fluke, *Fasciola hepatica* and the causative agent of Chagas disease, *Trypanosoma cruzi* [70–73]. In addition, antioxidant production in parasites has been linked to improved parasite survival [74, 75] and helminths are known to possess an array of ROS-reducing products, such as superoxide dismutase (SOD) [76] with *A*. *suum* also possessing catalase (CAT) [77], and peroxiredoxin (Prx) [76, 78], which offer the nematode mechanisms for ROS contention.

An intrinsic difference between the two mouse strains was also observed for glutathione metabolism, with C57BL/6J mice displaying a higher abundance in proteins involved in this pathway. Under infection, both strains displayed an increase in abundance for these proteins, with C57BL/6J mice presenting a more pronounced upregulation. Most mitochondria lack CAT, therefore, H_2_O_2_ conversion is maintained by glutathione (GSH) within these organelles [79]. H_2_O_2_ is reduced to H_2_O by glutathione peroxidase (GPx), using GSH [80] and this reaction produces oxidised glutathione (GSSG), which is then reduced back to GSH by glutathione reductase (GR), consuming one molecule of NADPH. This increase would need to be countered by increased antioxidants, such as GSH.

Our findings clearly indicate that mitochondrial- and ROS-associated proteins are more abundantly expressed in both strains under infection, indicative of the host liver being within a state of stress. In C57BL/6J mice, these increases are less pronounced than in CBA/Ca mice indicating that although a response to *Ascaris* is mounted, the levels achieved in C57BL/6Js are not equivalent to those in CBA/Ca mice. In addition, because ROS levels are presumably reduced in C57BL/6J mice, the nematodes entering the liver have a less cytotoxic environment to contend with and can potentially mount immuno-modulatory and antioxidative perturbations on their host.

### A more pronounced immune response is observed in C57BL/6J mice

The host immune system represents an important obstacle to the completion of the parasite life-cycle. To circumvent this threat, parasites have evolved strategies to evade or modulate aspects of the host immune response. In relation to *Ascaris*, previous studies examining the host immune response within the liver have focussed on the role of pro-inflammatory cytokines in co-ordinating defences against the parasite during its migratory phase [31, 81, 82]. Within the present study, gene ontology and KEGG pathway analyses failed to identify any clear process level immune responses to *Ascaris* infection and few differentially abundant immune proteins were identified either between strains or in response to the presence of *Ascaris*. The low number of immune-responsive proteins may be explained in two ways. Firstly, the liver is an immune tolerant organ and the hepatic system circumvents the lymphatic system. This ensures that no unwanted immune activation occurs in response to commensal pathogens present in the bowel, which can sometimes enter the blood stream after endothelial damage [83]. This system is thought to be exploited by other parasites, such as malaria-causing *Plasmodium* species, where the parasite enters the liver in the pre-erythrocytic stage, potentially to evade the host immune system [84]. Secondly, the experimental time point examined within the present study may be too early in the infection to observe an adaptive immune response, as the adaptive immune response takes 4–7 days to engage fully [85]. Of the identified differentially abundant immune proteins, broadly three groups can be defined (see Table 2): proteins observed in both mouse strains, protein abundance only observed in C57BL/6J mice, and proteins observed in CBA/Ca mice only. Overall, there appeared to be more immune-associated proteins that had increased abundance in response to infection, which was to be expected, and C57BL/6J mice displayed a higher abundance in some immune-associated proteins.

The proteins observed to be more abundant in both strains signify a potential innate immune response through the identification of proteins, such as complement component C8a and S100 proteins, S100a8 and S100a9. The alpha polypeptide of complement 8 interacts with other complement proteins to form the membrane attack complex, a cell-killing structure [86]. The complex binds to the cell membrane of target cells, forming transmembrane channels resulting in cell lysis and subsequent death [86]. Although, complement recruitment can occur in response to tissue damage potentially associated with parasite infection, complement components have previously been implicated as factors in mediating the adherence of myeloid cells to nematode parasites, resulting in parasite death although the susceptibility amongst different nematode species is variable [87]. Secretory products released by nematodes, such as *Brugia malayi* and *Trichinella spiralis*, have been identified to inhibit the chemotaxis activities of complement component 5a, identifying the complement component as a target for immunomodulation by parasitic nematodes [88].

Proteins S100a8 and S100a9 were among the most abundantly expressed under infection in both mouse strains, and completely absent in the control samples (Fig 1C and 1D). The S100 group is involved in multiple cellular functions, including cellular contraction, motility, cell differentiation and calcium regulation [89]. S100a8 and S100a9 can exist as homodimers, but in the presence of calcium, they will form a heterodimer called calprotectin [90, 91] which is expressed mainly by granulocytes, monocytes, and macrophages [90]. These proteins were recently identified as danger-associated molecular patterns (DAMPs) [92] which have been shown to interact with toll-like receptors (TLRs), such as TLR4 [93]. It is thought that S100a8/S100a9 are actively released by cells sensing danger, rather than passively [90]. Calprotectin has been found to be a chemotactic factor for neutrophils and other mononuclear cells, with the exception of lymphocytes, in mice [94], further confirming its role in the immune system. S100a8 and S100a9 heterodimers are known to be important in *Leishmania* infection. S100a8 and S100a9 recruitment results in the elimination of *Leishmania* and knockout mice experiencing a more severe infection. Such proteins probably have a role in the recruitment of neutrophils to the site of infection [91]. Moreover, S100a8 and S100a9 primed macrophages were better at killing *Leishmania* than non-primed macrophages. In addition, Edgeworth *et al*. [95], using human biopsies, found that S100a8/S100a9 heterodimer secretion, by macrophages, onto adults of the filarial nematode, *Onchocerca volvulus*, was part of an early immune response—demonstrating a putative immune role against nematode parasites. A similarly early response may be signified by the presence of these proteins in infected C57BL/6J and CBA/Ca mice suggesting a conserved role in defence against parasitic nematodes. In short, our results suggest the presence of an innate immune response (see Table 2) through the presence of a complement protein (C8a) and several proteins involved in leukocyte chemotaxis (such as S100a8, S100a9, galactin-3). Additionally, there is evidence of an early adaptive immune response through the presence of proteins such as plastin-2 (Lcp1) and dipeptidyl peptidase 1 (Ctsc).

Given that C57BL/6J mice carry more active *Ascaris* than CBA/Ca mice, it was unsurprising to find that a higher number of immune-associated proteins were observed in the susceptible strain. Of the nineteen proteins with known immune function (Table 2) fifteen had higher fold changes in C57BL/6J compared to CBA/Ca mice or were exclusively more statistically abundant in C57BL/6J mice. The two most abundantly expressed proteins in the ‘C57BL/6J’ only group, were annexin A1 and neutrophil gelatinase-associated lipocalin, both of which are expressed as a cellular response to interleukin-1 (IL-1). It seems therefore that the restrictions encountered by the nematode larva in the resistant strain results in the reduced immune response in comparison to their susceptible counterparts.

### Reduction in the translation machinery occurs under infection

Very little is known about how extracellular macroparasites, such as *Ascaris*, successfully modulate their host. The results of this study suggest that under infection, a down regulation of translational proteins occurs for both strains, in particular S6 ribosomal protein (part of the mammalian/mechanistic target of rapamycin (mTOR) pathway). Additionally, CBA/Ca control mice had, both with and without infection, higher ribosomal protein abundances compared to C57BL/6J mice. Under infection this intrinsic difference, however, became more pronounced. Pathogens are known to interact with translational proteins in their host [96]. Viruses, for example, are dependent on the host translational machinery for their own protein synthesis. Cells in turn can promote gene expression in response to the environmental situation, e.g. hypoxia, glucose deprivation [96] and pathogens, such as certain bacteria, are known to secrete effectors into the cytoplasm which in turn can reduce translation itself [97]. Inactivation of translation machinery is a documented strategy of certain parasites, such as the trypansome, *Leishmania major* [98]. Once the parasite has invaded a macrophage, the surface protease GP63 cleaves mTOR and mTOR complex 1 (mTORC1), which inhibits translational initiation [98]. It is reasonable to postulate that the widespread lower abundance of translation proteins may indicate direct modulation by *Ascaris* which is known to secrete a wide range of effector-like molecules [69]. However it must be acknowledged that the down regulation of translation may in fact signify a defensive strategy employed by the host, in response to damage caused by *Ascaris*, as is seen in bacteria-induced epithelial damage, which results in the triggering of signals that suppress host translation [96].

### Haemoglobin profiles distinguish both strains

Aged erythrocytes are broken down by macrophages in the spleen and liver [99]; the liver is therefore an important organ in haemoglobin metabolism. Haemoglobin beta-2 (Hbb2) is the most abundantly expressed protein in CBA/Ca mice, and displayed relative fold changes of over 12,500 between CBA/CA and C57BL/6J controls and a fold chance of over 500 between CBA/CA and C57BL/6J infected mice. The haemoglobin of mice consists of two haemoglobin alpha (Hbba), one haemoglobin beta-1 (Hbb-1, major chain) and one Hbb-2 (minor chain) subunits. There are three different haplotypes for haemoglobin beta (Hbb): HbbS (single, β^S^), HbbD (diffuse, β^D^) and HbbP. C57BL/6J mice are homozygous for HbbS [100], whereas CBA mice are heterozygous for HbbD [100–102]. The β^S^ haplotype has one reactive cysteine residue on position 93 (βCys93), whereas β^D^ and β^P^ have an extra reactive cysteine residue: βCys13 [100]. Interestingly, both βCys93 and βCys13 can be modified by GSSG, with βCys13 being more susceptible than βCys93 [100]. Furthermore, GSSG may be reduced to GSH at βCys13, without the need of GR nor NADPH [100]. Hempe *et al*. [100] postulate that the concentration of these cysteine sulfhydryl groups determines the availability of GSH for enzymatic reactions. Having a higher concentration of haemoglobin could therefore be a mechanism of CBA/Ca mice to establish their ROS tolerance (as observed in the previously mentioned radiation studies), as a higher haemoglobin concentration would coincide with more βCys93, and thus more chances for glutathione to be reduced. The allelic differences in haemoglobin are also thought to confer a differential ability of various mouse strains to contend with different parasites. For example, C57 mice (HbbS) are relatively resistant to *Plasmodium* infection compared to BALB/c mice (HbbD)[103].

### Conclusion

Ascariasis is a debilitating disease affecting an estimated 800 million individuals globally. While the pathology of the disease has been extensively examined, our understanding of the molecular mechanisms underlying resistance and susceptibility to nematode infection is poorly understood. Here we provide a novel insight into the changes in a host liver proteome in response to *Ascaris* infection *in vivo* within two murine strains varying in their resistance and susceptibility to infection. Our results provide evidence for significant intrinsic differences in the hepatic proteomes of both mouse strains, potentially associated with resistance to *Ascaris* infection. Given the higher levels of proteins associated with ROS producing and processing and the general increased tolerance to ROS in CBA/Ca mice [59] in comparison to C57BL/6J mice, in particular, we hypothesise that higher ROS levels and the associated oxidative environment could be involved in the inhibition of *Ascaris* larval in CBA/Ca mice. Whether the intrinsic differences in mitochondrial protein abundances are due to different levels of mitochondrial biogenesis and number between the two strains has yet to be determined. Our research provides new insights into the intricacies and complexities of the host-parasite relationship of *Ascaris*. In addition, potential parasite modulation of translational processes by *Ascaris* were clearly evident in both strains. Our findings also provide a new understanding of previous studies that utilised these two mouse strains for experiments involving early-onset hearing loss, radiation exposure, and several other micro and macroparasite infections. Given our findings and the central role of the liver in the *Ascaris* migratory pathway, we suggest a potentially novel research direction to develop alternative preventative control strategies for *Ascaris*. It seems that the key determinant in murine resistance to *Ascaris* lies in highly oxidative conditions that presumably restricts and arrests successful larval migration within the CBA/Ca hepatic environment. Larval nematodes that enter the C57BL/6J liver seem free to continue their onward progression and through the secretion of their excretory/secretory compounds further sustain their parasitic lifecycle through manipulation and modulation of the host liver. So although defence responses are mounted in C57BL/6J mice it seems that *Ascaris* is already well-established in its attempts to contend with the host response. However, through the manipulation of hepatic ROS levels in the susceptible mouse strain, we may now be able to determine the importance of intrinsic ROS in conferring resistance to *Ascaris*. Although significant research is required to fully understand the determinants of resistance to *Ascaris* in our murine model, it does seem that we have at least been presented with new options in our pursuit of strategies to control a disease that affects an estimated one eighth of our planet’s population.

### UniProt accession numbers for proteins mentioned in text

7-alpha-hydroxycholest-4-en-3-one 12-alpha-hydroxylase: O88962; ADP-ribosylation factor 5: P84084; Aldehyde oxidase 3: G3X982; Annexin A1: P10107; Chitinase-3-like protein 3: O35744; Collagen alpha-1(I) chain: P11087; Complement C4-B: P01029; Complement component C8 alpha chain: Q8K182; Coronin-1A: O89053; Costars family protein ABRACL: Q4KML4; Cytochrome c oxidase subunit 7A2, mitochondrial: P48771; Cytochrome c oxidase subunit 7C, mitochondrial: P17665; Dipeptidyl peptidase 1: P97821; Galectin-3: P16110; H-2 class I histocompatibility antigen K-B alpha chain: P01901; H-2 class I histocompatibility antigen, D-B alpha chain: P01899; H-2 class I histocompatibility antigen, K-B alpha chain: P01901; Haemoglobin alpha: P01942; Haemoglobin beta-1: P02088; Haemoglobin subunit beta-2: P02089; Histone H1.1: P43275; Histone H1.4: P43274; Interleukin-1 beta: P10749; Mitochondrial antiviral-signalling: Q8VCF0; Moesin: P26041; Murinoglobulin-2: P28666; Neutrophil gelatinase-associated lipocalin: P11672; Plasminogen: P20918; Plastin-2: Q61233; Protein S100-A8: P27005; Protein S100-A9: P31725; Putative hydroxypyruvate isomerase: Q8R1F5; S6 ribosomal protein: P62754; Seminal vesicle secretory protein 4: P18419; S-methylmethionine: Q91WS4; Tyrosine aminotransferase: Q8QZR1; Vitronectin: P29788. UniProt accession numbers are provided for all identified proteins in S1 and S2 Datasets.

## Supporting Information

S1 Dataset Proteins identified from the liver left lobes of C57BL/6J and CBA/Ca mice with and without *Ascaris* infection. (XLSX)

S2 Dataset Statistically significantly differentially abundant proteins (2 sample t-tests; p<0.05) and relative fold change differences for comparisons of C57BL/6J infected to C57BL/6J control, CBA/Ca infected to CBA/Ca control, CBA/Ca control to C57BL/6J control and C57BL/6J infected to CBA/Ca infected samples. (XLSX)

S1 Table Exclusively expressed proteins in C57BL/6J infected, C57BL/6J control, CBA/Ca infected and CBA/Ca control mice. Only proteins exclusive to or absent from all three replicates in a given sample are reported. A zero log_2_ normalised intensity value indicates a protein that was absent or undetected in a sample.
(XLSX)

S2 Table Fisher exact test for enrichment in Uniprot Keywords GOBP, GCC and KEGG terms. Fisher exact test with a Benjamini-Hochberg corrected FDR of 2%, on t-test significant differential abundant and exclusively expressed proteins from C57BL/6J and CBA/Ca mice with and without *Ascaris* infection. NTD: No term determined.
(XLSX)

S1 Fig Interaction network analysis of up regulated proteins in CBA/Ca and C57BL/6J control and infected mice. Protein interaction information was obtained from the STRING database using gene lists extracted for statistically significant differentially abundant (SSDA) proteins from pair wise t-tests (p< 0.05). Each node represents a protein and each connecting line represents an interaction, the extent of evidence for which is represented by the width of the line. Statistically enriched KEGG and biological process Gene Ontology (GO) descriptors were examined to identify clusters of proteins enriched within SSDA protein of increased abundance for **(A)** CBA/Ca control in comparison to C57BL/6J control, **(B)** C57BL/6J control in comparison to CBA/Ca control, **(C)** CBA/Ca infected in comparison to C57BL/6J infected and **(D)** C57BL/6J infected in comparison to CBA/Ca infected mice.
(TIF)

S2 Fig KEGG analysis displaying significantly changing enzymes involved in the process of oxidative phosphorylation and the components of the ribosome in CBA/Ca and C57BL/6J control and infected mice. **(A)** Differentially expressed proteins involved in oxidative phosphorylation. CBA/Ca control and infected mice display a higher abundance of enzymes (red, purple and cyan) compared to C57BL/6J control and infected mice (blue, orange and pink). A significant proportion of these proteins have higher abundances in CBA/Ca control mice with respect to their C57BL/6J counterparts, indicating a clear intrinsic difference between the strains. (**B)** Differentially expressed proteins involved with the ribosome. C57BL/6J control samples had less ribosomal proteins than their CBA/Ca control counterparts (blue and red) indicative of an innate difference between these two strains. Under infection this intrinsic difference is also observed, with higher abundance for ribosomal proteins in CBA/Ca mice (purple and orange) observed.
(TIF)

S3 Fig KEGG analysis displaying significantly changing enzymes involved in the TCA Cycle in CBA/Ca and C57BL/6J control and infected mice. **(A)** Differentially expressed proteins involved in the TCA cycle in control mice. A clear intrinsic difference is observed between CBA/Ca (red) and C57BL/6J mice (blue). **(B)** Differentially expressed proteins involved in the TCA cycle in infected mice. Under infection C57BL/6J mice display a higher abundance of TCA cycle proteins (yellow).
(TIF)

S4 Fig KEGG analysis displaying significantly changing enzymes involved in glutathione metabolism in CBA/Ca and C57BL/6J control and infected mice. Differentially expressed proteins involved glutathione metabolism. Glutathione metabolism enzymes are more abundant in C57BL/6J control mice (blue) and compared to their CBA/Ca counterparts indiacting an intrinsic difference between the strains. Under infection C57BL/6J mice had increased expression of glutathione metabolism proteins (blue and cyan) and increase that was less pronounced in CBA/Ca *Ascaris*-infected mice (red).
(TIF)
