# Supplementary figures and images for: A Proteomic Investigation of Hepatic Resistance to Ascaris in a Murine Model
Source: PLoS Negl Trop Dis. 2016 Aug 4;10(8):e0004837. doi: 10.1371/journal.pntd.0004837 (PMC4974003; doi:10.1371/journal.pntd.0004837)

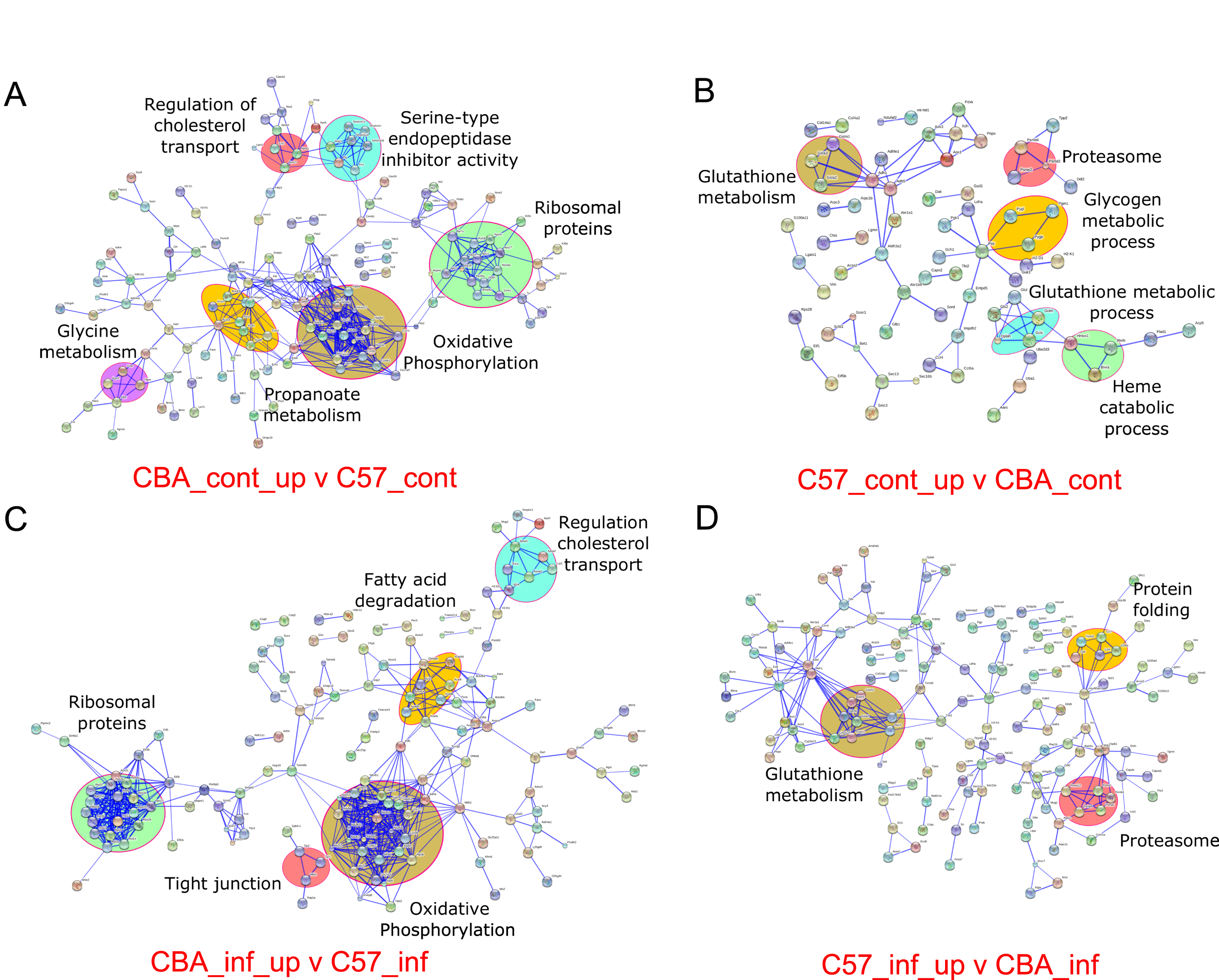

Supplement: S1 Fig — Protein interaction information was obtained from the STRING database using gene lists extracted for statistically significant differentially abundant (SSDA) proteins from pair wise t-tests (p< 0.05). Each node represents a protein and each connecting line represents an interaction, the extent of evidence for which is represented by the width of the line. Statistically enriched KEGG and biological process Gene Ontology (GO) descriptors were examined to identify clusters of proteins enriched within SSDA protein of increased abundance for (A) CBA/Ca control in comparison to C57BL/6J control, (B) C57BL/6J control in comparison to CBA/Ca control, (C) CBA/Ca infected in comparison to C57BL/6J infected and (D) C57BL/6J infected in comparison to CBA/Ca infected mice. (TIF) [file pntd.0004837.s005.tif]

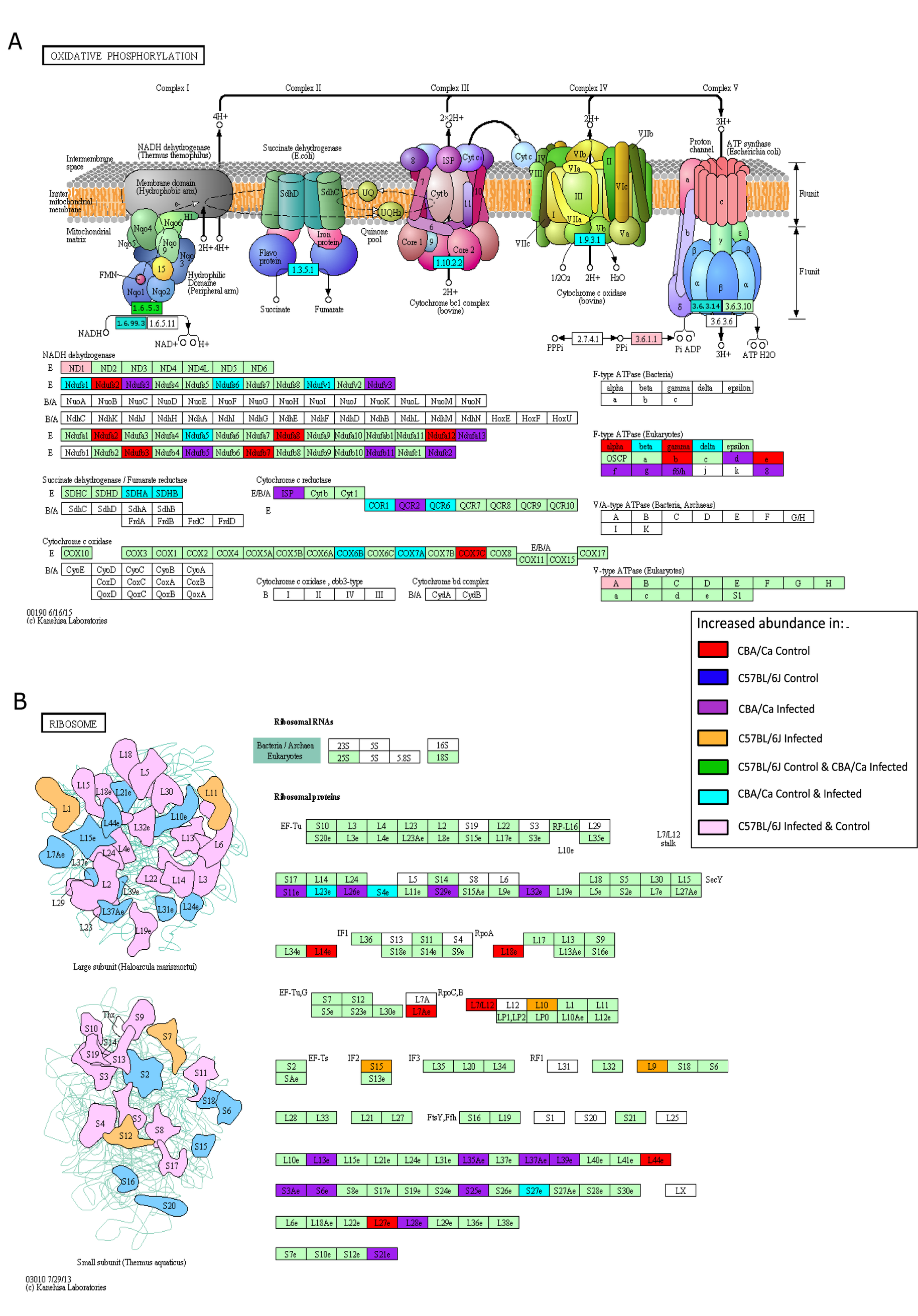

Supplement: S2 Fig — (A) Differentially expressed proteins involved in oxidative phosphorylation. CBA/Ca control and infected mice display a higher abundance of enzymes (red, purple and cyan) compared to C57BL/6J control and infected mice (blue, orange and pink). A significant proportion of these proteins have higher abundances in CBA/Ca control mice with respect to their C57BL/6J counterparts, indicating a clear intrinsic difference between the strains. (B) Differentially expressed proteins involved with the ribosome. C57BL/6J control samples had less ribosomal proteins than their CBA/Ca control counterparts (blue and red) indicative of an innate difference between these two strains. Under infection this intrinsic difference is also observed, with higher abundance for ribosomal proteins in CBA/Ca mice (purple and orange) observed. (TIF) [file pntd.0004837.s006.tif]

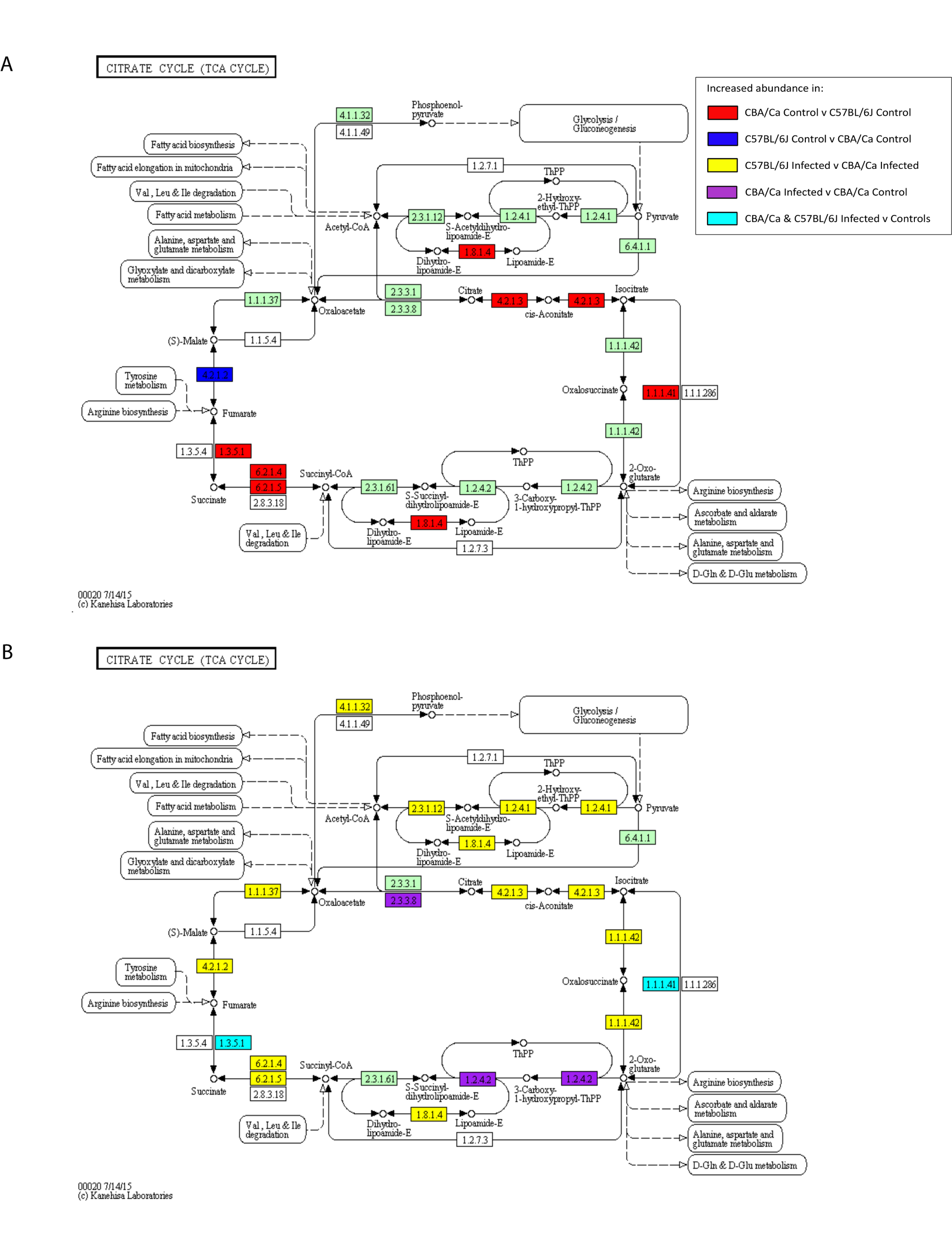

Supplement: S3 Fig — (A) Differentially expressed proteins involved in the TCA cycle in control mice. A clear intrinsic difference is observed between CBA/Ca (red) and C57BL/6J mice (blue). (B) Differentially expressed proteins involved in the TCA cycle in infected mice. Under infection C57BL/6J mice display a higher abundance of TCA cycle proteins (yellow). (TIF) [file pntd.0004837.s007.tif]

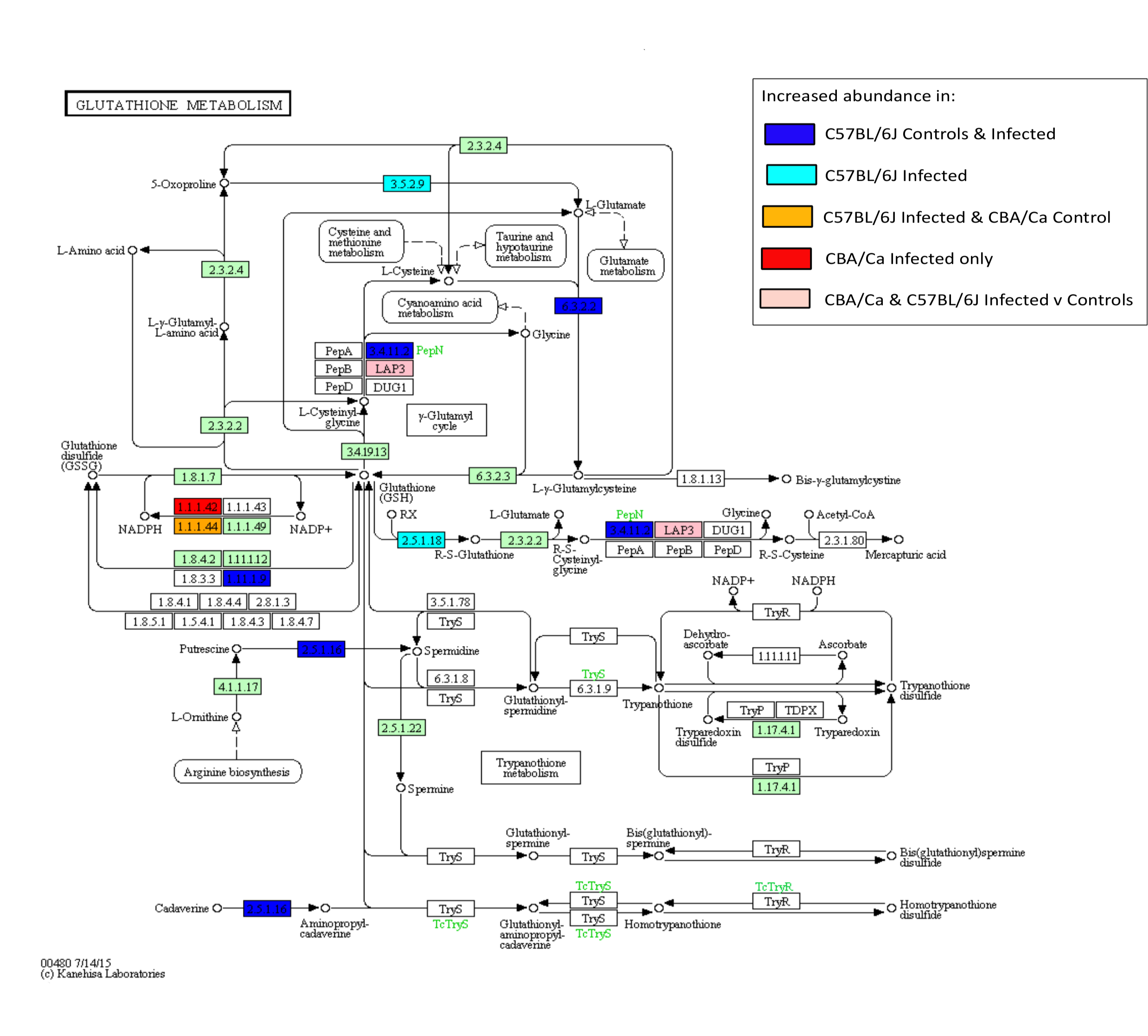

Supplement: S4 Fig — Differentially expressed proteins involved glutathione metabolism. Glutathione metabolism enzymes are more abundant in C57BL/6J control mice (blue) and compared to their CBA/Ca counterparts indiacting an intrinsic difference between the strains. Under infection C57BL/6J mice had increased expression of glutathione metabolism proteins (blue and cyan) and increase that was less pronounced in CBA/Ca Ascaris-infected mice (red). (TIF) [file pntd.0004837.s008.tif]
